# A Feed-Forward Loop Coupling Extracellular BMP Transport and Morphogenesis in *Drosophila* Wing

**DOI:** 10.1371/journal.pgen.1003403

**Published:** 2013-03-21

**Authors:** Shinya Matsuda, Jorge Blanco, Osamu Shimmi

**Affiliations:** Institute of Biotechnology, University of Helsinki, Helsinki, Finland; University of Pennsylvania School of Medicine, United States of America

## Abstract

A variety of extracellular factors regulate morphogenesis during development. However, coordination between extracellular signaling and dynamic morphogenesis is largely unexplored. We address the fundamental question by studying posterior crossvein (PCV) development in *Drosophila* as a model, in which long-range BMP transport from the longitudinal veins plays a critical role during the pupal stages. Here, we show that RhoGAP Crossveinless-C (Cv-C) is induced at the PCV primordial cells by BMP signaling and mediates PCV morphogenesis cell-autonomously by inactivating members of the Rho-type small GTPases. Intriguingly, we find that Cv-C is also required non-cell-autonomously for BMP transport into the PCV region, while a long-range BMP transport is guided toward ectopic wing vein regions by loss of the Rho-type small GTPases. We present evidence that low level of ß-integrin accumulation at the basal side of PCV epithelial cells regulated by Cv-C provides an optimal extracellular environment for guiding BMP transport. These data suggest that BMP transport and PCV morphogenesis are tightly coupled. Our study reveals a feed-forward mechanism that coordinates the spatial distribution of extracellular instructive cues and morphogenesis. The coupling mechanism may be widely utilized to achieve precise morphogenesis during development and homeostasis.

## Author Summary

It has been extensively studied how tissue morphogenesis is regulated by a variety of extracellular cues. Given that dynamic morphogenesis coincides with arrival of extracellular factors, there must be also mechanisms that coordinate extracellular signaling and intracellular morphogenesis. However, the coordination is largely unknown, due to the complexity of morphogenesis *in vivo*. We addressed the fundamental question by studying posterior crossvein (PCV) development in *Drosophila* as a model, in which a long-range transport of bone morphogenetic protein (BMP) type ligands from adjacent longitudinal veins plays a critical role during the pupal stages. Here, we first showed that RhoGAP Crossveinless-C (Cv-C) is induced at the PCV region by BMP signal and mediates PCV morphogenesis. By modulating wing vein morphogenesis, we then found that PCV morphogenesis is required for BMP transport, while ectopic wing vein morphogenesis sufficiently guides a long-range BMP transport. These data suggest a feed-forward mechanism that coordinates the spatial distribution of extracellular instructive cues and morphogenesis. The coupling mechanism may be widely utilized to achieve precise tissue morphogenesis and tissue homeostasis.

## Introduction

A key question in developmental biology is to address how tissue morphogenesis is regulated by a variety of extracellular signals. This includes identification of such extracellular signaling molecules and intracellular mechanisms that trigger morphogenesis. Since arrival of extracellular factors coincides with dynamic morphogenesis, there must be mechanisms to coordinate signaling and morphogenesis. The coordination can be achieved by an instructive role of morphogenesis in determining the regions where extracellular signals arrive or are activated. However, this is largely unknown, due to the complexity of morphogenesis *in vivo*.

The bone morphogenetic proteins (BMPs) are extracellular factors that regulate morphogenesis as well as growth and patterning [1], [2]. In *Drosophila*, Decapentaplegic (Dpp), a homologue of BMP2/4, is secreted either as a homodimer or a heterodimer with another BMP-type ligand (Glass bottom boat (Gbb) or Screw (Scw)). The ligands bind to the type I receptor Thickveins (Tkv) and type II receptor Punt and phosphorylate the transcription factor Mad. Then phosphorylated Mad (pMad), together with Medea, translocates into the nucleus for transcriptional regulation of various genes [3]. The nuclear accumulation of pMad can be visualized by immunostaining and used as a readout of the BMP signal.

The Rho-type small GTPases, including Rho, Rac, and Cdc42, play critical roles in actin cytoskeleton organization, cell-extracellular matrix (ECM) adhesion, cell polarity, cell cycle progression, and cell migration [4], [5]. The activities of the Rho-type small GTPases are tightly regulated by the guanine nucleotide exchange factors (GEFs) and GTPase-activating proteins (GAPs). The GEFs activate the GTPases by replacing GDP with GTP, while the GAPs inactivate the GTPases by enhancing their GTP-hydrolyzing activity [6], [7]. Recent studies have shown that BMP signaling regulates epithelial morphogenesis through the Rho-type small GTPases. However, transcriptional downstream factors that link the BMP signal with the activities of the Rho-type small GTPases are largely unknown [8], [9].

**Figure 1:**
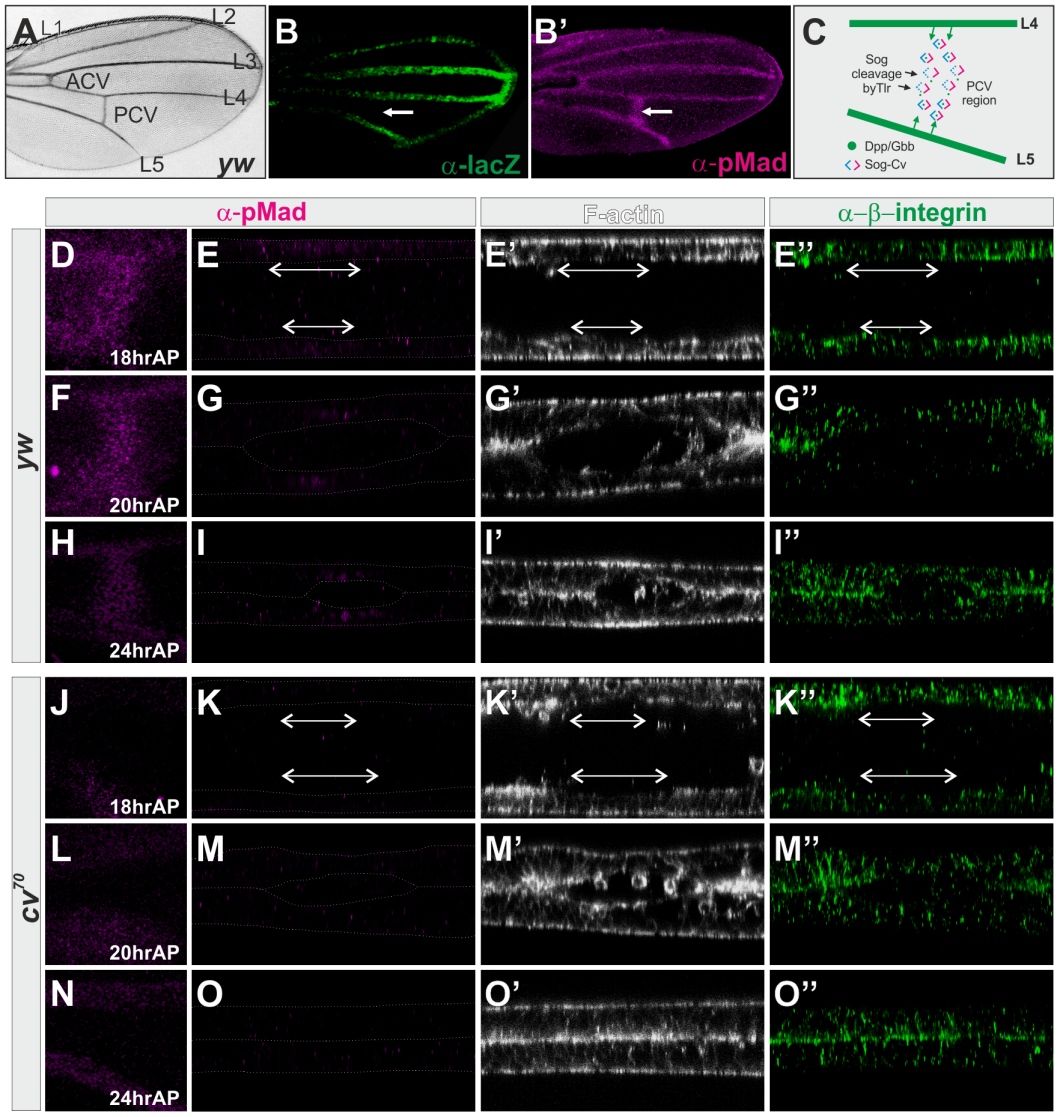
BMP signaling is required for PCV morphogenesis. (A) Wild-type *yw* adult wing. (B, B′) lacZ (B) and pMad (B′) staining of *dpp^shv^-lacZ* at 24 hr AP. The PCV position is indicated by arrows. (C) A current model of Sog-Cv-mediated directional BMP transport from the LVs into the PCV. (D—I) Wild-type *yw* pupal wing. pMad (D, E, F, G, H, I), F-actin (E′, G′, I′), and ß-integrin (E″, G″, I″) staining at 18 hr AP (D, E), 20 hr AP (F, G), and 24 hr AP (H, I). (J—O) *cv^70^* pupal wing. pMad (J, K, L, M, N, O), F-actin (K′, M′, O′), and ß-integrin (K″, M″, O″) staining at 18 hr AP (J, K), 20 hr AP (L, M), and 24 hr AP (N, O). (D, F, H, J, L, N) Dorsal view of the PCV region. (E, G, I, K, M, O) Optical cross-sections of the PCV region. Prospective PCV positions are indicated by double-headed arrows at 18 hr AP (E–E″, K–K″).

Posterior crossvein (PCV) development mediated by BMP signaling during the pupal stages provides an excellent system for understanding how the long-range BMP signal regulates morphogenesis (Figure 1A, 1B). *dpp* is initially transcribed at the prospective longitudinal veins (LVs) (Figure 1B), then later also in the PCV region about 28 hr after pupariation (AP) [10]. In contrast, the BMP signal is detected at all the vein primordia from about 17—18 hr AP (Figure 1B′). It has been thus proposed that Dpp diffuses from the adjacent LVs (L4 and L5) toward the PCV region during 18—28 hr AP for PCV development [10]–[12]. By visualizing Dpp distribution in the pupal wing, we recently demonstrated that the Dpp-Gbb heterodimer is directionally transported from the LVs into the PCV region through two BMP-binding proteins, Short gastrulation (Sog) and Crossveinless (Cv) (Figure 1C) [13]. Cleavage of Sog by the protease Tolloid-related (Tlr) then releases the ligands to activate the receptors (Figure 1C) [12]. Interestingly, the direction of BMP transport or PCV position is prefigured independently of BMP signaling by lack of *sog* transcription at the PCV region about 20 hr AP [10], [13], which is thought to help generate the Sog gradient that guides BMP towards the PCV region [13]. To date, the BMP signal mediated by the BMP transport is the earliest instructive signal for PCV formation. A similar BMP transport mechanism also operates in the patterning of the early embryo [14]–[16].

In contrast with the extracellular regulation of BMP transport, little is known about how the BMP signal regulates wing vein morphogenesis recognized by apposition at the basal side of two wing epithelial layers [17]. Furthermore, directional BMP transport toward the PCV region undergoing morphogenesis raises a question of how BMP transport and wing vein morphogenesis are coordinated [13].

A candidate for mediating PCV formation is Crossveinless-C (Cv-C), whose viable mutant allele displays a PCV-less phenotype [18]. Recently, *cv-c* was identified as RhoGAP88C required for a variety of embryo morphogenesis [19]. However, it remains unclear how Cv-C regulates PCV formation. Here, we show that *cv-c* is induced at the PCV region by the BMP signal and mediates PCV morphogenesis by inactivating various members of the Rho-type small GTPases. Intriguingly, we found that loss of *cv-c* inhibited Sog-Cv dependent BMP transport into the PCV region, while an ectopic Sog-Cv-dependent BMP signal was induced toward ectopic wing veins by loss of the Rho-type small GTPases. Taken together, our data suggest that Cv-C mediates a feed-forward loop coupling BMP transport and PCV morphogenesis. We also provide evidence that the initial PCV morphogenesis precedes BMP signaling and *sog* transcription, highlighting an instructive role of morphogenesis in guiding BMP transport.

## Results

### BMP signaling is required for PCV morphogenesis

To investigate how the PCV region undergoes morphogenesis, we analyzed optical cross-sections in the prospective PCV region, marked by pMad accumulation. The tissue architecture and the cell-extracellular matrix (ECM) adhesion were visualized by phalloidin staining of F-actin and immunostaining for ß-integrin [20]. At 18 hr AP, two wing epithelial layers were separated with the similar tissue architecture between the PCV region and intervein regions (Figure 1E). Around 20—21 hr AP, the wing vein lumen was formed through apposition of the basal side of the intervein regions. The apical-basal cell length in the PCV region became shorter than that in the intervein regions (Figure 1G). At 24 hr AP, the apposition continued except the PCV region (Figure 1I) and apical-basal polarity is maintained between the PCV region and intervein regions (Figure S6A–S6A‴). During 18—24 hr AP, F-actin and ß-integrin preferentially accumulated at the basal side of the intervein epithelial cells, but less at the basal side of the PCV region (Figure 1E′, 1E″, 1G′, 1G″, 1I′, 1I″). F-actin also accumulated at the apical side of the wing epithelial cells, which became more evident at the apical side of the PCV region at 24 hr AP (Figure 1I′). Ubiquitous expression of ß-integrin in the pupal wing suggests that the ß-integrin distribution is regulated posttranscriptionally (Figure S1). The lack of apposition of the two wing epithelial layers and the distinct tissue architecture in the PCV region recognized by less distribution of the ß-integrin and F-actin at the basal side are hereinafter referred to as PCV morphogenesis. We note that similar ß-integrin and F-actin distributions were observed in the LV formation [20].

Since PCV morphogenesis overlapped with the pMad accumulation (Figure 1D–1I), we investigated whether BMP signal is required for PCV morphogenesis. To test this, PCV morphogenesis was analyzed in *cv^70^*, a null allele of *cv*, in which pMad accumulation was absent due to lack of BMP transport (Figure 1J, 1K, 1L, 1M, 1N, 1O) [11], [13]. We found that, despite the smaller lumen size, the initial PCV morphogenesis occurred during 18—20 hr AP and was subsequently disrupted around 24 hr AP (Figure 1K, 1M, 1O). PCV morphogenesis was also analyzed in a *dpp^shv^* mutant (*dpp^s4^/dpp^s11^*) [21]. In *dpp^s4^/dpp^s11^*, BMP signal was severely affected both in the LVs and CVs during pupal stages (20–26 hr AP), and consequently, distal parts of L4, L5 and PCV were not formed in the adult wing (Figure S2A–S2C, data not shown) [13]. We found that PCV morphogenesis occurred during 22–24 hr AP and was disrupted until 26 hr AP in *dpp^s4^/dpp^s11^* (Figure S2D–S2G). These observations suggest that BMP signaling is not required for the initiation but for the maintenance of PCV morphogenesis through regulation of ß-integrin and F-actin localizations.

### The initial PCV morphogenesis is independent of *sog* transcriptional prepattern

**Figure 2:**
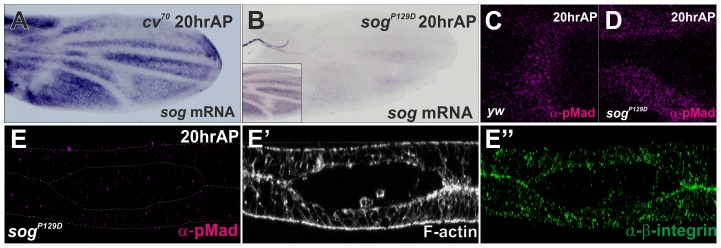
The initial PCV morphogenesis is independent of *sog* transcriptional prepattern. (A) *in situ* hybridization of *sog* at 20 hr AP in *cv^70^* pupal wing. (B) *in situ* hybridization of *sog* at 20 hr AP in *sog^P129D^* and in wild-type *yw* pupal wing (inset). (C–D) pMad staining at PCV region at 20 hr AP in *yw* (C) and in *sog^p129D^* (D). (E–E″) Optical cross-sections of the PCV region. pMad (E), F-actin (E′), and ß-integrin (E″) staining at 20 hr AP in *sog^P129D^*.

It has been shown that *sog* transcription also prepatterns the PCV position (Figure 2A) [10], [13]. To test whether the initial PCV morphogenesis is dependent on *sog* transcription, the initial PCV morphogenesis was analyzed at 20 hr AP in *sog^P129D^*, where *sog* transcriptional prepattern information and pMad signal were severely absent at the PCV region (Figure 2B–2D). We found that the initial PCV morphogenesis still occurred in *sog^P129D^* (Figure 2E–2E″). Thus, the initial PCV morphogenesis is independent of *sog* transcription. The initial PCV morphogenesis as well as *sog* transcription may be involved in guiding the BMP transport.

### Cv-C mediates PCV morphogenesis by inactivating various Rho-type small GTPases

**Figure 3:**
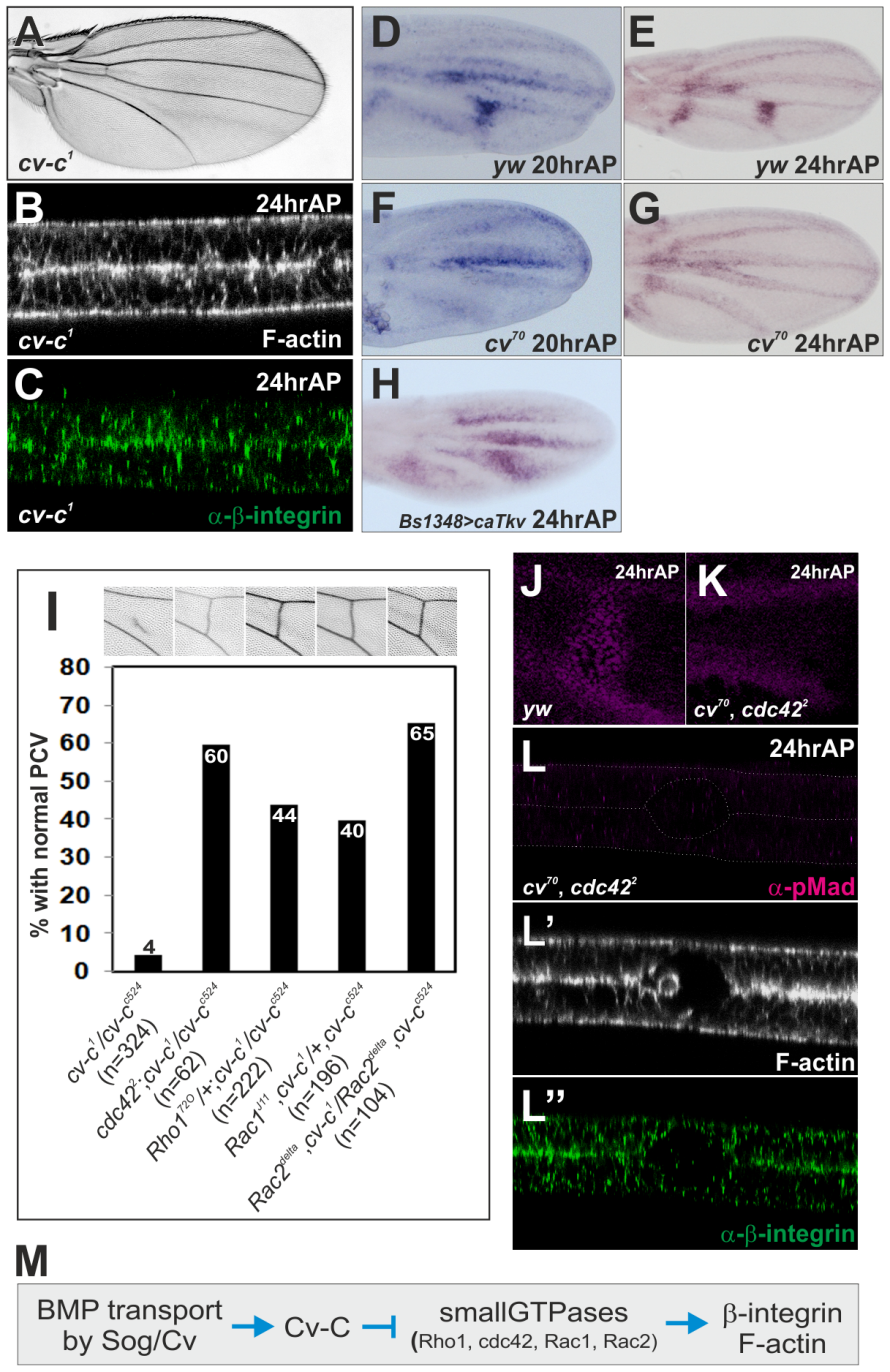
Cv-C mediates PCV morphogenesis downstream of BMP signaling by inactivating various Rho-type small GTPases. (A) Adult wing of *cv-c^1^*. (B, C) Optical cross-sections of the PCV region. F-actin (B) and ß-integrin (C) staining at 24 hr AP in *cv-c^1^*. (D—H) *in situ* hybridization of *cv-c* at 20 hr AP (D) and at 24 hr AP (E) in wild-type *yw*, and at 20 hr AP (F) and at 24 hr AP (G) in *cv^70^*, and at 24 hr AP in *BS1348>caTkv* (H). *BS1348-Gal4* is intervein-specific Gal4 driver. (I) Genetic interactions of *cv-c* with various small GTPases. Representative adult wings around the PCV region of each genotype are shown above. (J, K) pMad staining at 24 hr AP in wild-type *yw* (J) and in *cv^70^, cdc42^2^* double mutant (K). (L—L″) Optical cross-sections of the PCV region. pMad (L), F-actin (L′), and ß-integrin (L″) staining at 24 hr AP in *cv^70^, cdc42^2^* double mutant. (M) A schematic pathway regulating PCV morphogenesis downstream of BMP signaling.

We then asked what mediates PCV morphogenesis downstream of BMP signaling. A candidate for mediating PCV morphogenesis is RhoGAP Cv-C (Figure 3A), which regulates a variety of embryo morphogenesis through regulation of the cytoskeleton [19], [22]. Indeed, optical cross-sections showed defects in PCV morphogenesis at 24 hr AP in *cv-c^1^*, a viable allele of *cv-c* (Figure 3B, 3C). During 20—24 hr AP, *cv-c* is strongly expressed in the CVs and weakly in the LVs and intervein regions along the LVs (Figure 3D, 3E). Since *cv-c* expression is absent from the PCV region during 20—24 hr AP in *cv^70^* (Figure 3F, 3G) and ectopically induced by the constitutively active form of type I receptor Tkv (caTkv) at 24 hr AP (Figure 3H), *cv-c* transcription in the PCV region is tightly regulated by BMP signaling.

We then asked if Cv-C regulates PCV morphogenesis by inactivating the Rho-type small GTPases. In this case, adult PCV defects in *cv-c* may be rescued by reducing the activities of Rho-type small GTPases. Indeed, we found that severe adult PCV defects in *cv-c^1^/cv-c^c524^* were efficiently restored by mutant alleles of *Cdc42, Rho1, Rac1*, or *Rac2* (Figure 3I). Consistently, when Rho1 activity was visualized by a GFP-based sensor that binds to the active form of Rho1 in the dorsal wing layer [23], Rho1 activity was low in the PCV region and high at the basal side of the intervein region at 24 hr AP (Figure S3A). In contrast, GFP alone was uniformly distributed in the dorsal wing layer at 24 hr AP (Figure S3B). The Rho1 protein also less accumulated at the basal side of the PCV region at 24 hr AP (Figure S3D). The similar distribution of Rho1 activity was also observed in the dorsal wing layer at 20 hr AP in *cv^70^*, even though *cv-c* was not expressed in the PCV region (Figure 3F, Figure S3C). This suggests that Rho1 activity in the initial PCV morphogenesis is regulated independently of BMP signaling and Cv-C.

We then asked if inactivation of the Rho-type small GTPases is critical for PCV morphogenesis downstream of BMP signaling. In this case, defects in PCV morphogenesis due to lack of BMP signaling may be rescued by reducing activities of Rho-type small GTPases. Indeed, by using the viable *cdc42* allele *cdc42^2^* [24], we found that defects in PCV morphogenesis in *cv^70^* (Figure 1N, 1O) were efficiently rescued at 24 hr AP in *cv^70^, cdc42^2^* double mutant independently of pMad signal (Figure 3J–3L). Taken together, these data suggest that Cv-C is induced in the PCV region by BMP signaling and regulates ß-integrin and F-actin distribution by inactivating various Rho-type small GTPases (Figure 3M).

### Cv-C is non-cell-autonomously required for BMP signaling

**Figure 4:**
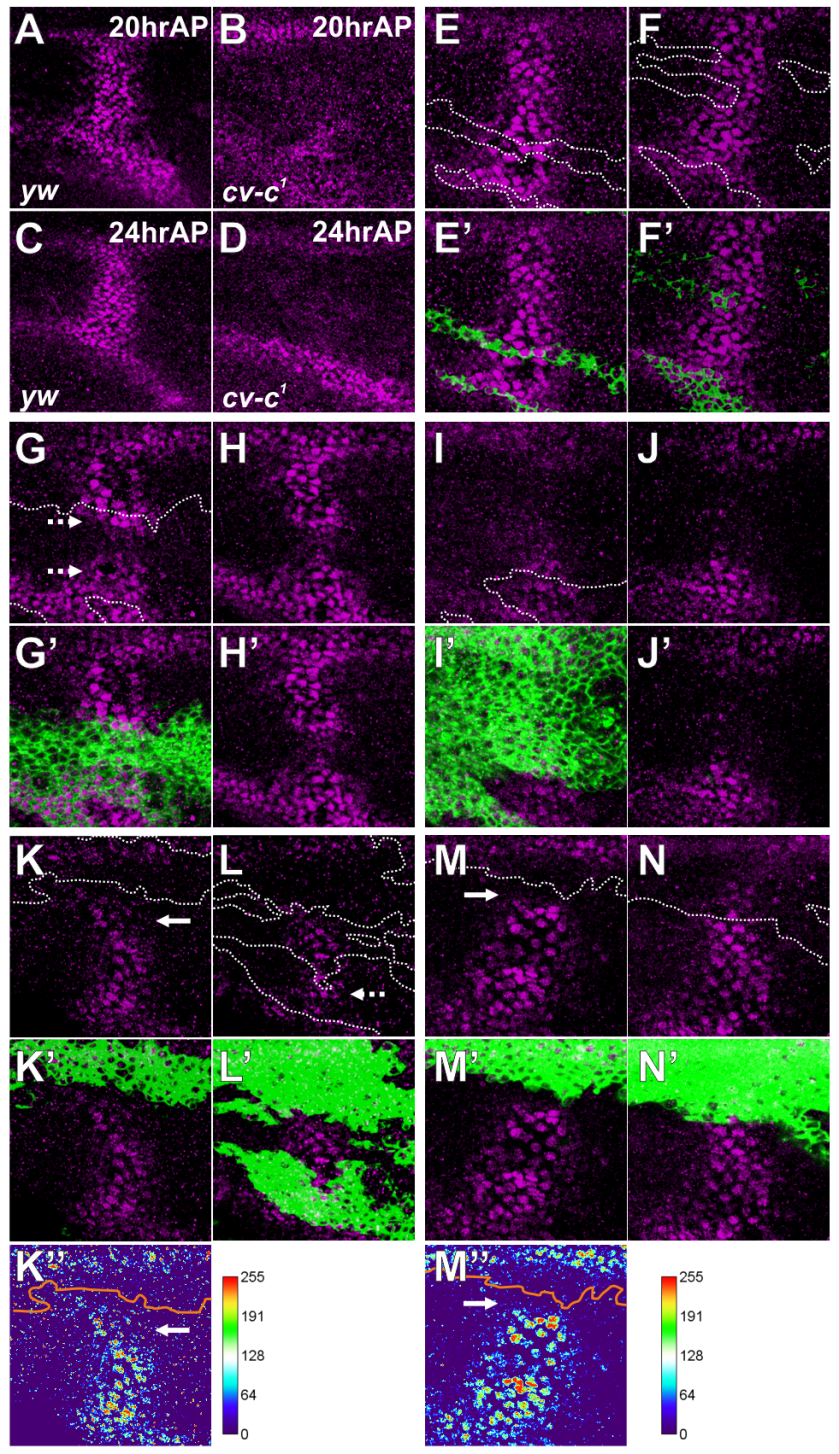
Cv-C is non-cell-autonomously required for BMP signaling. (A—D) pMad staining at 20 hr AP (A, B) and at 24 hr AP (C, D) in wild-type *yw* (A, C) and *cv-c^1^* (B, D). (E—N) Effect of homozygous *cv-c^c524^* clones (green, E′—N′) on pMad (purple, E—N) at 24 hr AP. (E–J, M, N) A single confocal image from different wing layers of the same wing (E—F, G—H, I—J, and M—N). (K, L) Maximum intensity projections from a single wing layer of the same wing. Clone boundaries were marked by white line in E—N and by orange line in K″, M″. Mutant cells accumulating pMad signal in the PCV region are marked by dashed arrows (G, L). Wild-type cells lacking pMad accumulation in the PCV region are marked by arrows (K, M).

Unexpectedly, we found that loss of *cv-c* affects BMP signaling in the PCV region. In *cv-c^1^*, pMad signal in the PCV region was almost absent during 20–24 hr AP (Figure 4A–4D). To investigate how Cv-C is involved in BMP signaling, mutant clones of *cv-c^c524^*, a null allele of *cv-c*, were generated using the MARCM system [25]. Since BMP ligands are produced in the LVs of both wing layers and diffuse into the PCV region to activate BMP signal in both layers (Figure 1) [13], we analyzed the effects of *cv-c* mutant clones on BMP signal in both layers. We found that pMad accumulation appeared normal within small mutant clones (Figure 4E, 4F), suggesting that *cv-c* mutant cells can activate BMP signal. In such *cv-c* mutant clones, the apical-basal cell length became longer with F-actin accumulation at the basal side (Figure S4). In contrast, when mutant clones covered about half of the PCV region in one wing layer, pMad accumulation was normal in a few mutant cells adjacent to wild-type cells but attenuated in the middle of the clones (Figure 4G). When mutant clones straddled almost all the PCV region in one wing layer, pMad signal was severely affected within clones (Figure 4I). Interestingly, in these cases, pMad signal was also attenuated in the PCV region in the other wing layer (Figure 4H, 4J). When mutant clones in both layers were overlapped at the PCV region, pMad signal was more effectively inhibited within overlapping double-sided clones (Figure 4K–4N) than single-sided mutant clones (dashed arrows Figure 4G, 4L), and was sometimes lost even in the wild-type cells adjacent to mutant clones (arrows in Figure 4K, 4K″, 4M, 4M″). These results indicate that Cv-C is non-cell autonomously required for BMP signaling in the PCV region. The non-autonomous effects of *cv-c* mutant clones on BMP signal across the wing layer (Figure 4H, 4J, 4K, 4M) indicate that BMP ligands transported into one wing layer activate BMP signal in both wing layers. BMP ligands derived from the wild-type wing layer probably cross the lumen to activate BMP signal within *cv-c* mutant cells or wild-type cells between single-sided clones (Figure 4G, 4L).

### Cv-C is required for Sog-Cv-dependent BMP transport into the PCV region

**Figure 5:**
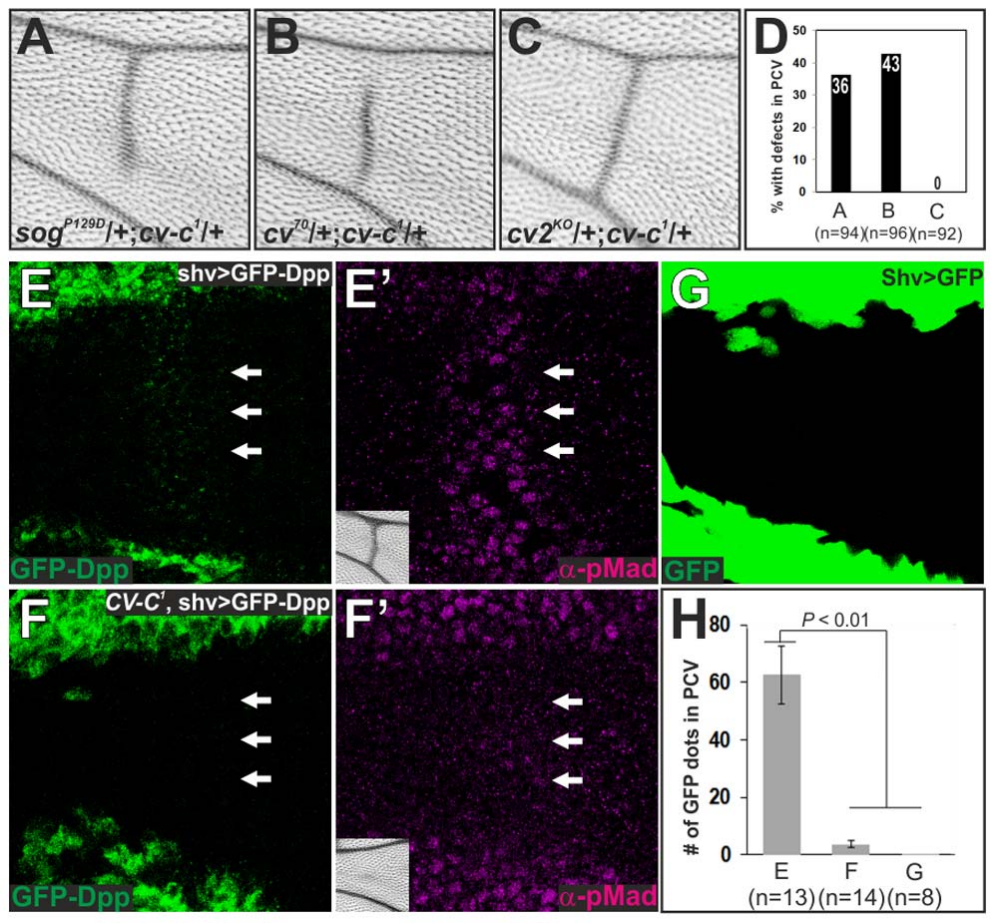
Cv-C is required for Sog-Cv-dependent BMP transport into the PCV region. (A–C) Genetic interaction of *cv-c* with *sog* (A), *cv* (B), or *cv-2* (C). Representative adult wings around the PCV region of each genotype are shown. (D) Quantification of adult wing phenotypes in A–C. (E, F) GFP-Dpp distribution (E, F) and pMad staining (E′, F′) at 24 hr AP and adult PCV region (inset) in wild-type *yw* (*shv>GFP-Dpp*) (E, E′) and in *cv-c* (*cv-c^1^*, *shv>GFP-Dpp*) (F, F′). The PCV position is indicated by arrows. (G) GFP distribution at 24 hr AP in *shv>GFP*. Despite strong GFP signal in LVs, no dots were observed in the PCV region. (H) Quantification of the number of GFP dots in E, F and G. Statistical significance was determined using Mann-Whitney U test.

The non-cell-autonomous function of Cv-C on BMP signaling can be mediated through BMP transporters or BMP-binding protein Crossveinless-2 (Cv-2), which facilitates ligand-receptor binding in a short-range manner [13], [26]. We found partial PCV defects in wings transheterozygous for *cv-c* and *sog*, or for *cv-c* and *cv*, but not for *cv-c* and *cv-2* (Figure 5A–5D). The genetic interaction between Cv-C and BMP transporters suggests that Cv-C is involved in BMP transport toward the PCV region. To test this, we visualized Dpp distribution in the PCV region by expressing functional *GFP*-*dpp* in the LVs [13], [27]. In the wild-type background, GFP-Dpp dots accumulated at the PCV region at 24 hr AP, where pMad signal is positive (62.5±10.1 dots, n = 13 wings) (Figure 5E, 5H). In contrast, GFP-Dpp dots and pMad accumulation were significantly reduced at the PCV region at 24 hr AP (3.6±1.2 dots, n = 14 wings), and consequently the adult PCV defect was not rescued in *cv-c^1^* mutant background (Figure 5F, 5H). When GFP was expressed in the LVs, GFP dots did not accumulate at the PCV region (0.1±0.1 dots, n = 8 wings) (Figure 5G, 5H). Taken together, these data indicate that Cv-C is required for Sog-Cv-dependent BMP transport into the PCV region.

### Sog-Cv-dependent BMP signaling is induced at the ectopic wing veins by loss of the Rho-type small GTPases

**Figure 6:**
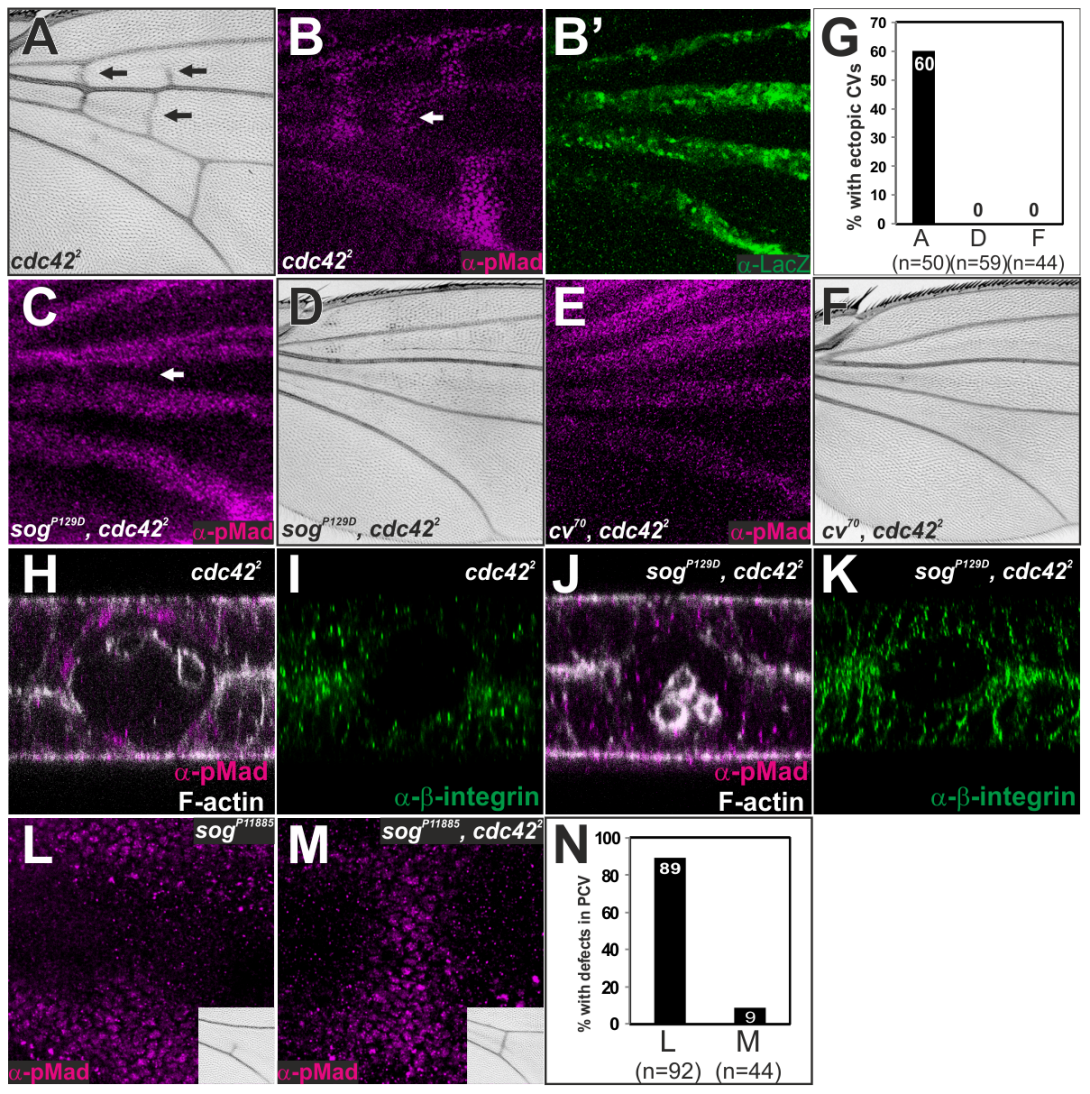
Sog-Cv-dependent BMP signaling is induced along ectopic wing vein morphogenesis by loss of *cdc42*. (A) Adult wing of *cdc42^2^*. (B, B′) pMad (B) and lacZ staining (B′) in *cdc42^2^* ; *dpp^shv^-lacZ/+*. (C—F) pMad staining (C, E) and adult wing (D, F) of *sog^P129D^, cdc42* ^2^ (C, D) and *cv^70^*, *cdc42* ^2^ (E, F). (G) Quantification of the phenotypes in A, D and F. (H—K) Optical cross-sections of the ectopic CV region (white arrows in B and C). pMad and F-actin (H, J) and ß-integrin (I, K) staining in *cdc42^2^* (H, I) and *sog^P129D^*, *cdc42* ^2^ (J, K). The relative ß-integrin accumulation at the basal side of the ectopic CV region by comparing to that of intervein regions is 32±2% (n = 7 wings) in *cdc42^2^* (I) and 37±3%, (n = 6 wings) in *sog, cdc42* (K). (L, M) pMad staining and adult PCV (inset) in *sog^P11885^* (L) and in *sog^P11885^, cdc42^2^* (M). (N) Quantification of the adult wing phenotypes in L and M. All pupal wings were fixed at 24 hr AP.

How does Cv-C regulate BMP transport? Since *cv-c* is required for PCV morphogenesis, we hypothesized that BMP transport is coupled with wing vein morphogenesis. To test this, we analyzed *cdc42^2^*, in which ectopic CVs were frequently observed in the adult wings (Figure 6A, 6G) [28], [29]. We found that ectopic pMad signal was induced in the future ectopic CVs at 24 hr AP in *cdc42^2^* without changing *dpp* transcription (Figure 6B, 6B′). The ectopic pMad signal and CVs formation in *cdc42^2^* were completely cancelled in *sog^P129D^*, *cdc42^2^* or *cv^70^, cdc42^2^* double mutant (Figure 6C–6G). Optical cross-sections revealed that ectopic pMad signal was detected at the ectopic wing vein regions (arrow in Figure 6B) at 24 hr AP in *cdc42^2^* (Figure 6H, 6I). We found that the ectopic wing vein morphogenesis occurred independently of pMad signal in the corresponding region (arrow in Figure 6C) in *sog^P129D^*, *cdc42^2^* double mutant (Figure 6J, 6K). These data indicate that Sog-Cv-dependent BMP transport was guided toward ectopic wing veins by loss of *cdc42*. Sog-Cv-dependent ectopic pMad accumulation and ectopic adult wing veins were also induced by loss of *Rho1* (Figure S5). We note that BMP signaling independent wing vein morphogenesis at 24 hr AP by loss of *cdc42* (Figure 3L, Figure 6J and 6K) never induced adult wing veins (Figure 6D, 6F, 6G), suggesting that additional factors are required to form adult wing veins downstream of BMP signal after 24 hr AP.

We then addressed whether PCV morphogenesis plays an instructive role in BMP transport. In this case, despite loss of pMad signal in *cv^70^, cdc42^2^*, or *sog^P129D^*, *cdc42^2^* double mutant due to lack of BMP transport (Figure 6C, 6E), defects in pMad signal in weak alleles of BMP transporters may be rescued by reducing the activities of the Rho-type small GTPases. Indeed, we found that defects of pMad and adult PCV in *sog^p11885^*, a weak hypomorphic allele of *sog*, were efficiently restored in *sog^p11885^*, *cdc42^2^* double mutant (Figure 6L–6N). These results suggest that Sog-Cv-mediated BMP transport is tightly coupled with wing vein morphogenesis by loss of the Rho-type small GTPases.

### ß-integrin links Sog-Cv-dependent BMP signaling and PCV morphogenesis

**Figure 7:**
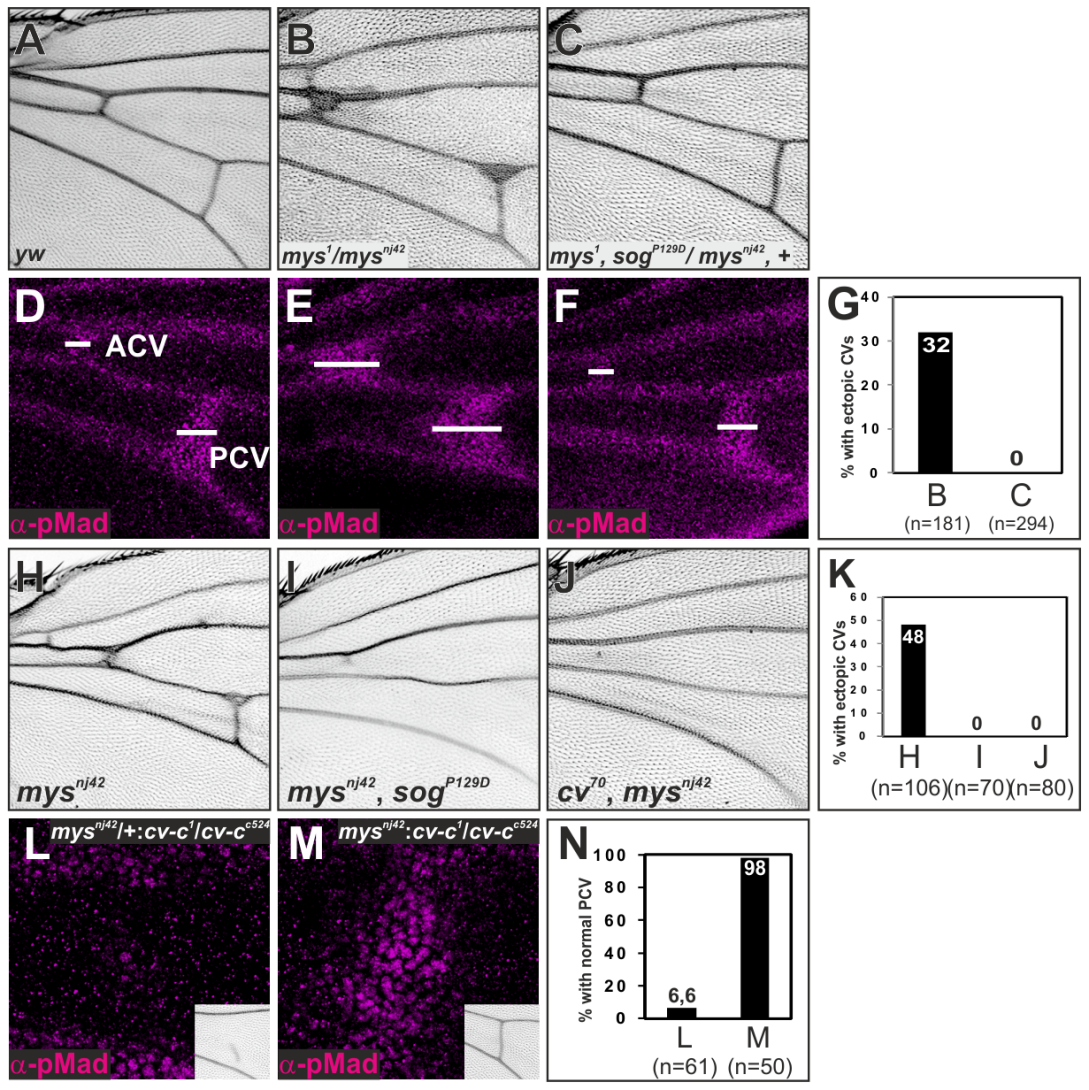
ß-integrin links Sog-Cv-dependent BMP signaling and PCV morphogenesis. (A—F) Adult wings (A—C) and pMad staining (D—F) of wild-type *yw* (A, D), *mys^1^/mys^nj42^* (B, E), and *mys^1^, sog^P129D^/mys^nj42^*, + (C, F). (G) Quantification of adult wing phenotypes in B and C. (H—J) Adult wings of *mys^nj42^* (H), *mys^nj42^, sog^p129D^* (I), and *cv^70^, mys^nj42^* (J). (K) Quantification of adult wing phenotype in H—J. (L, M) pMad staining at 24 hr AP and adult PCV (insets) in *mys^nj42^/+; cv-c^1^/cv-c^c524^* (L) and *mys^nj42^*; *cv-c^1^/cv-c^c524^* (M). (N) Quantification of adult wing phenotype in L and M.

What is the molecular mechanism that couples BMP transport and wing vein morphogenesis? We found that ectopic pMad accumulation by loss of *cdc42* or *Rho1* was associated with low ß-integrin at the basal side of wing epithelial cells (Figure 6H–6K, Figure S5). Integrins have been previously proposed to regulate Sog protein distribution from the intervein regions into the LVs during the pupal stages [30]. This raises the possibility that integrins may link Sog-Cv-dependent BMP transport and PCV morphogenesis. Indeed, we found that ectopic adult wing veins including ectopic or thickened CVs were induced in the ß-integrin *myospheroid* (*mys*) mutants, *mys^1^/mys^nj42^*or *mys^nj42^* (Figure 7A, 7B, 7H) [30], [31], in a *sog*- or *cv*-dependent manner (Figure 7A–7C, 7G–7K). Although anterior crossvein (ACV) development requires BMP transport [11], wing vein fragments were often observed at the ACV region in *mys^nj42^, sog^P129D^* or *cv^70^, mys^nj42^* double mutant (Figure 7I, 7J), which may reflect *dpp* expression in a part of ACV region during pupal stages [10]. Ectopic pMad accumulation at the PCV region was also observed in *mys^1^/mys^nj42^* in a *sog*-dependent manner (Figure 7D–7F). In fact, quantification of GFP-Dpp distributions along apical-basal axis in wings expressing GFP-Dpp in the LVs showed accumulation of GFP-Dpp dots at 21±2% (n = 125 dots, 3 wings) from the basal surface in the PCV region (Figure S6E), where ß-integrin is less distributed (Figure 1). Furthermore, we found that pMad and adult PCV defects in *cv-c^1^/cv-c^c524^* were efficiently rescued in *mys^nj42^*, *cv-c^1^/cv-c^c524^* double mutants (Figure 7L–7N). This indicates that low ß-integrin activity promotes BMP transport at the basal side of the PCV region downstream of Cv-C. Taken together, these data suggest that BMP transport is coupled with PCV morphogenesis through ß-integrin.

## Discussion

Despite the critical roles of BMP signaling in various morphogenetic processes, little is known about its transcriptional downstream factors that mediate morphogenesis. Using *Drosophila* PCV morphogenesis as a model, we found that RhoGAP Cv-C is induced by BMP signaling and mediates PCV morphogenesis through inactivation of various Rho-type small GTPases. Furthermore, we found that Cv-C is required non-cell-autonomously for BMP transport into the PCV region, while BMP signaling is induced at the ectopic wing veins by loss of the Rho-type small GTPases. These results suggest that Cv-C mediates a feed-forward loop coupling BMP transport and PCV morphogenesis.

How does the activity of Rho-type small GTPases control morphogenesis in the pupal wing? Recent studies have revealed that cellular compartmentalization of the Rho-type small GTPases is critical to regulate epithelial morphogenesis [9], [23], [32]. We found a similar biased distribution of Rho1 activity in the pupal wing. Rho1 activity is high at the basal side of the intervein regions and low in the PCV region (Figure S3). Since overexpression of constitutively active form of Rho1 as well as Rho1 RNAi shortens apical-basal cell lengths in the intervein region [33], localized Rho1 activity rather than total activity is associated with the apical-basal cell length. Localized activities of Rho-type small GTPases then regulate ß-integrin and F-actin accumulation at the basal side (Figure 6H–6K) and maintain apical-basal cell length in the intervein region probably by mediating cell-ECM adhesion as in the larval wing epithelium [34]. In the PCV region, we showed that Cv-C is induced by BMP signal and inactivates various Rho-type small GTPases to induce shorter apical-basal lengths (Figure 3). Interestingly, BMP signaling has been previously linked with the localized Rho1 activity that triggers apical-basal cell elongation in the larval wing imaginal disc [9]. Thus, BMP signaling may positively or negatively regulate the compartmentalization of Rho-type small GTPases in a tissue-dependent manner.

**Figure 8:**
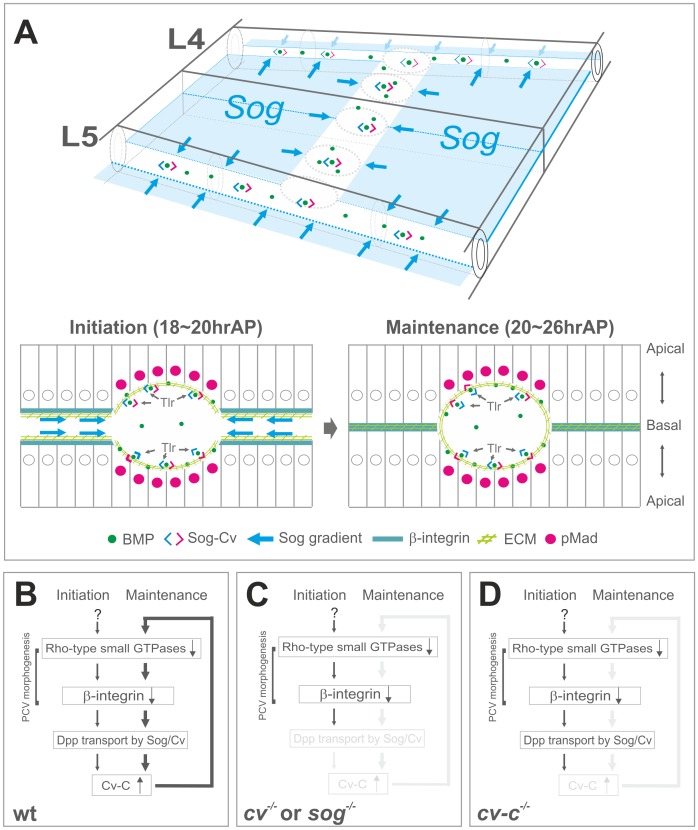
A model of coupling of BMP transport and PCV morphogenesis. (A) Schematic overview of BMP transport and cross-section in the PCV region. BMP transport appears to occur through the ECM along PCV morphogenesis. (B) In wild-type pupal wing, the initial PCV morphogenesis is induced by inactivation of Rho-type small GTPases independently of BMP signaling. BMP transport is then guided toward the PCV region through low ß-integrin activity (∼18 hr AP). BMP signaling induces RhoGAP Cv-C to maintain PCV morphogenesis and BMP transport through a feed-forward loop (20∼26 hr AP). (C) In *cv* or *sog* pupal wing, the initial PCV morphogenesis occurs but is not maintained, due to the absence of BMP transport. (D) In *cv-c* pupal wing, BMP signaling and PCV morphogenesis are not maintained without a feed-forward loop.

Our study revealed that RhoGAP Cv-C also regulates BMP transport through PCV morphogenesis (Figure 6, Figure 7). How then is BMP transport coupled with wing vein morphogenesis? Our data showed that GFP-Dpp is basally accumulated at the PCV region (Figure S6) [13]. Furthermore, a recent study showed that vittelogenin-like protein Crossveinless-d (Cv-d) is secreted from hemocytes to regulate BMP signaling in the PCV region [35]. These observations suggest that BMP is transported into the PCV region through the lumen (Figure 8) [13]. The lumen may provide physical space for BMP transport. However, pMad accumulates in the PCV region even before the lumen is formed (Figure 1). We found that BMP transport is rather associated with ß-integrin distribution (Figure 1, Figure 6, Figure 7). Since Sog-Cv dependent BMP signal is induced in the ß-integrin-free regions adjacent to the LVs (Figure S5), low level of ß-integrin activity guides BMP transport from the LVs probably by affecting Sog distribution [30]. Since integrins physically interact with Sog [30], we presume that the Sog protein gradient is formed along the ECM to direct Sog-Cv-BMP complex towards the PCV region (Figure 8). BMPs are then released from Sog-Cv by Tlr and activate the signal in the PCV region on the both wing layers. In this model, BMP transport is effectively blocked when *cv-c* mutant clones are overlapped in both layers (Figure 4K–4N). In contrast, when *cv-c* mutant clones are generated in one wing layer, BMP transport is attenuated in *cv-c* mutant cells, but BMP ligands can be supplied from the other wing layer to activate BMP signal in *cv-c* mutant cells (dashed arrows Figure 4G, 4L). However, since total amounts of BMP ligands are reduced in the PCV region, BMP signaling range is affected in both wing layers (Figure 4H, 4J).

How is Sog gradient established along the ECM? ECM components may help Sog distribution. Collagen IV has been recently shown to regulate BMP signal in a variety of developmental processes [36], [37]. In contrast with Collagen IV accumulation at the basal side of the larval wing imaginal disc (Figure S6D) [38], Collagen IV accumulated as few punctate spots at the basal side and in the hemocytes in the pupal wing (Figure S6A–S6C). Since punctate Collagen IV signal is randomly distributed at the basal side, Collagen IV may not be actively involved in BMP signal at the PCV region but probably degraded by hemocytes for remodeling the pupal wing [39]. Another ECM component Laminin has been shown to genetically interact with Sog [30]. Thus Laminin may be involved in regulating Sog distribution. Recent studies also identified novel extracellular factors that regulate BMP signal via heparan sulfate proteoglycans (HSPGs) [40], [41]. Among them, Pentagon (Pent) regulates BMP morphogen gradient via the glypican Dally in the larval wing imaginal discs [40]. Although loss of *pent* did not induce evident PCV defects, Dally and Dally-like are required for BMP signal at the PCV region non-cell autonomously [35]. Pentagon may be involved in PCV formation together with HSPGs.

Our results also provide insights into the positional information for the PCV formation. Since *sog* mutant could not induce evident ectopic CVs [11], [13] and PCV formation could be rescued in some cases despite ubiquitous *sog* expression [26], it has been argued that *sog* repression in the PCV region may not be sufficient to provide the positional information for the PCV development [10], [13]. We showed that the initial PCV morphogenesis provides prepattern information independently of *sog* transcription (Figure 2). Thus the initial *sog* transcription and ß-integrin localization may cooperate to establish Sog gradient to instruct BMP transport toward the PCV region. BMP transport is then maintained by a positive feedback mechanism through Cv-C (Figure 8). Although the factors that regulate the initial PCV morphogenesis remain to be elucidated, they may regulate Rho1 activity in the initial PCV region (Figure S3C, Figure 8).

Instructive role of morphogenesis for the cell specification was also recently reported in pancreatic tubulogenesis, where Cdc42 mediated tubulogenesis controls cell specification [42]. Our data further suggest the coupling of extracellular cues and dynamic morphogenesis via instructive role of morphogenesis. The coupling mechanism provides two general implications. First, the coupling mechanism ensures spatial distribution of secreted factors at the tissues undergoing dynamic morphogenesis without restricting the competence to respond to signaling. Indeed, the intervein regions can respond to BMP signaling [13]. Second, a positive feedback mechanism allows for continuous signaling to the target tissues. This would be especially important when continuous signaling is required for further differentiation. In fact, adult wing vein morphogenesis requires continuous BMP signaling (Figure 1O, Figure 6D and 6F).

In conclusion, we identified a Cv-C-mediated feed-forward mechanism that couples BMP transport and PCV morphogenesis. Given that BMP signaling and a human homolog of Cv-C, Deleted in liver cancer (DLC1), act as tumour suppressors in a variety of contexts [43], [44], a similar coupling mechanism may be operated in tissue homeostasis as well as tissue morphogenesis. As illustrated in our study, investigating simple *in vivo* models would provide further insights into the coordination between extracellular cues and dynamic morphogenesis.

## Materials and Methods

### Fly strains

The *cv^70^*, *sog^P129D^*, *cv2^KO1^*, *shv^3Kpn^*-Gal4, and *UAS-GFP-Dpp* flies were described previously [13]. The *cv-c^1^*and *cv-c^c524^* flies were obtained from H. Skaer [19]. The *UAS-PNKG58AeGFP* flies were kindly provided by J.C. Hombria [23]. The mys*^nj42^* flies were obtained from F. Schoeck. The *dpp^shv^-lacZ*, *dpp^s11^*, *dpp^s4^*, *cdc42^2^*, *Rho1^72O^*, *Rac2^Δ^*, *Rac1^J11^*, *UAS-GFP*, *sog^P11885^*, *UAS-Rho1RNAi*, *BS1348-Gal4*, and *mys^1^* flies were obtained from Bloomington Drosophila Stock Center. *Vkg-GFP* flies were obtained from Fly Trap stock collection. *cv-c* MARCM clones were generated using *y w hs-FLP*; *tubP-GAL4 UAS-mCD8::GFP*; *FRT82B cv-c^c524^*/*FRT82B tubP-GAL80*.

### Immunostaining and *in situ* hybridization

*Drosophila* pupal wings were fixed at 4°C overnight and then dissected from the pupae. All immunohistochemistry and *in situ* hybridizations were performed, as previously described [11]. The primary antibodies were as follows: rabbit anti-pMad at 1∶1000 (a gift from P. ten Dijke), mouse anti-LacZ at 1∶1000 (Promega), mouse anti-ß-integrin (CF.6G11) at 1∶100 (Developmental Studies Hybridoma Bank (DSHB)), rabbit anti-aPKC (C-20) at 1∶200 (Santa Cruz Biotechnology, Inc.), and mouse anti-Dlg (4F3) at 1∶100 (DSHB). The secondary antibodies were as follows: anti-rabbit IgG-Alexa 568 or 647 and anti-mouse IgG-Alexa 488 were used at 1∶1000, respectively (Invitrogen). Can Get Signal Solution B (TOYOBO) was used for staining with anti-pMad and anti-ß-integrin. The fluorescent images were obtained with a Leica TCS SP5 confocal microscope.

### Quantification of the images

To analyze the number of GFP-Dpp dots, approximately 10 confocal sections were taken at app. 1-µm intervals to cover a single cell layer and the images were processed by maximum intensity profile and quantified using analyze particle command in ImageJ software (National Institutes of Health, Bethesda, MD, USA). To analyze GFP-Dpp distribution along apical-basal axis, relative position of GFP-Dpp dots from basal surface of the PCV region (the basal surface is set to 0% and the apical surface is set to 100%) was measured individually in a single confocal image using Image J. The heatmap of the intensity of pMad signal was produced using the “HeatMap Histogram” plugin of ImageJ.

## Supporting Information

Figure S1 ß-integrin expression in the pupal wing. (A—D) *in situ* hybridization of ß-integrin at 20 hr AP (A, B) and at 24 hr AP (C, D). Antisense (A, C) and sense (B, D) probes of *myospheroid* (*mys*) encoding ß-integrin.
(TIF)

Figure S2 The initial PCV morphogenesis in *dpp^s4^/dpp^s11^*. (A) Adult wing of *dpp^s4^/dpp^s11^*. (B, C) pMad staining at 24 hr AP in control *yw* (B) and in *dpp^s4^/dpp^s11^* (C). (D–F) Optical cross-sections of the PCV region. F-actin staining at 22 hr AP (D), 24 hr AP (E), 26 hr AP (F) in *dpp^s4^/dpp^s11^*. A number of hemocytes were observed in the lumen at 22 hr AP. Slight delay of apposition of the two wing layers may reflect loss of the LVs fates in *dpp^s4^/dpp^s11^*. (G–G″) Optical cross-sections of the PCV region. pMad (G), F-actin (G′), and ß-integrin (G″) staining at 24 hr AP in *dpp^s4^/dpp^s11^*.
(TIF)

Figure S3 Rho1 activity and protein localization around the PCV region. (A) Rho1 activity in the dorsal layer at 24 hr AP in *ap>PKNG58AeGFP*. (B) GFP signal in the dorsal layer of *shv>GFP*. (C) Rho1 activity in the dorsal wing layer at 20 hr AP in *cv^70^*, *ap>PKNG58AeGFP*. (A, C) The basal side of the invervein regions is indicated by arrows. The PCV region is marked by parentheses. *ap-Gal4* is induced in the dorsal layer of the wing epithelial cells. (D) Rho1 (D), pMad (D′) staining, and merge (D″) in wild-type *yw*. All the images are optical cross-sections of the PCV region.
(TIF)

Figure S4 Cell-autonomous F-actin accumulation at the basal side of the PCV region in *cv-c^c524^* clones. A sagittal section of the PCV region, containing *cv-c* null clones. (A) *cv-c^c524^* clones (green), (A′) F-actin (white), and (A″) pMad (purple). F-actin accumulation at the basal side of the PCV is indicated by arrow. Apical-basal cell lengths of wild-type and *cv-c* mutant are indicated by double-headed arrows.
(TIF)

Figure S5 Loss of Rho1 by RNAi induces Sog-Cv-dependent BMP signaling. pMad (A, C, E), ß-integrin (A′, C′, E′) staining, merged images (A″, C″, E″), and adult wings (B, D, F) in wild-type *yw* (A—A″, B), *cv^70^*/+, *BS1348>Rho1 RNAi* (C—C″, D), and *cv^70^*, *BS1348>Rho1 RNAi*. (E—E″, F). The PCV position is indicated by arrows (A, C). Ectopic pMad accumulation is marked by arrowhead (C). All the pupal wings were fixed at 24 hr AP. Images were processed by maximum intensity profile from a single wing layer.
(TIF)

Figure S6 Apical-basal polarity in the PCV region and intervein region. (A–C) Optical cross-sections of the PCV region at 24 hr AP. (A) aPKC staining (A), Dlg staining (A′), Vkg-GFP signal (A″), and merged image (A‴) in *Vkg-GFP* wing. (B) Vkg-GFP signal (B) and F-actin staining (B′) in *Vkg-GFP* pupal wing. (C) GFP signal (C) and F-actin staining (C′) in control *yw* pupal wing. In addition to few punctuate signal, Collagen IV was also weakly and uniformly distributed in the lumen. (D) Vkg-GFP signal (D) and F-actin staining (D′) in *Vkg-GFP* larval wing imaginal disc. (E) Optical cross-sections of the PCV region at 24 hr AP. Representative images of GFP-Dpp distribution along apical-basal axis. GFP-Dpp dots (E), F-actin staining (E′), and schematic position of GFP-Dpp dots (E″) in *shv>GFP-Dpp*.
(TIF)

## References

[1] Affolter M, Basler K (2007) The Decapentaplegic morphogen gradient: from pattern formation to growth regulation. Nat Rev Genet 8: 663–674. 17703237 10.1038/nrg2166

[2] Wu MY, Hill CS (2009) Tgf-beta superfamily signaling in embryonic development and homeostasis. Dev Cell 16: 329–343. 19289080 10.1016/j.devcel.2009.02.012

[3] Parker L, Stathakis DG, Arora K (2004) Regulation of BMP and activin signaling in Drosophila. Prog Mol Subcell Biol 34: 73–101. 14979665 10.1007/978-3-642-18670-7_4

[4] Jaffe AB, Hall A (2005) Rho GTPases: biochemistry and biology. Annu Rev Cell Dev Biol 21: 247–269. 16212495 10.1146/annurev.cellbio.21.020604.150721

[5] Schwartz MA, Shattil SJ (2000) Signaling networks linking integrins and rho family GTPases. Trends Biochem Sci 25: 388–391. 10916159 10.1016/s0968-0004(00)01605-4

[6] Rossman KL, Der CJ, Sondek J (2005) GEF means go: turning on RHO GTPases with guanine nucleotide-exchange factors. Nat Rev Mol Cell Biol 6: 167–180. 15688002 10.1038/nrm1587

[7] Moon SY, Zheng Y (2003) Rho GTPase-activating proteins in cell regulation. Trends Cell Biol 13: 13–22. 12480336 10.1016/s0962-8924(02)00004-1

[8] Cordero JB, Larson DE, Craig CR, Hays R, Cagan R (2007) Dynamic decapentaplegic signaling regulates patterning and adhesion in the Drosophila pupal retina. Development 134: 1861–1871. 17428827 10.1242/dev.002972 PMC2957290

[9] Widmann TJ, Dahmann C (2009) Dpp signaling promotes the cuboidal-to-columnar shape transition of Drosophila wing disc epithelia by regulating Rho1. J Cell Sci 122: 1362–1373. 19366729 10.1242/jcs.044271

[10] Ralston A, Blair SS (2005) Long-range Dpp signaling is regulated to restrict BMP signaling to a crossvein competent zone. Dev Biol 280: 187–200. 15766758 10.1016/j.ydbio.2005.01.018

[11] Shimmi O, Ralston A, Blair SS, O'Connor MB (2005) The crossveinless gene encodes a new member of the Twisted gastrulation family of BMP-binding proteins which, with Short gastrulation, promotes BMP signaling in the crossveins of the Drosophila wing. Dev Biol 282: 70–83. 15936330 10.1016/j.ydbio.2005.02.029

[12] Serpe M, Ralston A, Blair SS, O'Connor MB (2005) Matching catalytic activity to developmental function: tolloid-related processes Sog in order to help specify the posterior crossvein in the Drosophila wing. Development 132: 2645–2656. 15872004 10.1242/dev.01838

[13] Matsuda S, Shimmi O (2012) Directional transport and active retention of Dpp/BMP create wing vein patterns in Drosophila. Dev Biol 366: 153–162. 22542596 10.1016/j.ydbio.2012.04.009

[14] Shimmi O, Umulis D, Othmer H, O'Connor MB (2005) Facilitated transport of a Dpp/Scw heterodimer by Sog/Tsg leads to robust patterning of the Drosophila blastoderm embryo. Cell 120: 873–886. 15797386 10.1016/j.cell.2005.02.009 PMC6460932

[15] Wang YC, Ferguson EL (2005) Spatial bistability of Dpp-receptor interactions during Drosophila dorsal-ventral patterning. Nature 434: 229–234. 15759004 10.1038/nature03318

[16] O'Connor MB, Umulis D, Othmer HG, Blair SS (2006) Shaping BMP morphogen gradients in the Drosophila embryo and pupal wing. Development 133: 183–193. 16368928 10.1242/dev.02214 PMC6469686

[17] Hogan BL, Kolodziej PA (2002) Organogenesis: molecular mechanisms of tubulogenesis. Nat Rev Genet 3: 513–523. 12094229 10.1038/nrg840

[18] Stern C (1934) On the Occurrence of Translocations and Autosomal Non-Disjunction in Drosophila melanogaster. Proc Natl Acad Sci U S A 20: 36–39. 16587837 10.1073/pnas.20.1.36 PMC1076335

[19] Denholm B, Brown S, Ray RP, Ruiz-Gomez M, Skaer H, et al (2005) crossveinless-c is a RhoGAP required for actin reorganisation during morphogenesis. Development 132: 2389–2400. 15843408 10.1242/dev.01829

[20] Fristrom D, Wilcox M, Fristrom J (1993) The distribution of PS integrins, laminin A and F-actin during key stages in Drosophila wing development. Development 117: 509–523. 8330522 10.1242/dev.117.2.509

[21] St Johnston RD, Hoffmann FM, Blackman RK, Segal D, Grimaila R, et al (1990) Molecular organization of the decapentaplegic gene in Drosophila melanogaster. Genes Dev 4: 1114–1127. 2120113 10.1101/gad.4.7.1114

[22] Brodu V, Casanova J (2006) The RhoGAP crossveinless-c links trachealess and EGFR signaling to cell shape remodeling in Drosophila tracheal invagination. Genes Dev 20: 1817–1828. 16818611 10.1101/gad.375706 PMC1522077

[23] Simoes S, Denholm B, Azevedo D, Sotillos S, Martin P, et al (2006) Compartmentalisation of Rho regulators directs cell invagination during tissue morphogenesis. Development 133: 4257–4267. 17021037 10.1242/dev.02588

[24] Fehon RG, Oren T, LaJeunesse DR, Melby TE, McCartney BM (1997) Isolation of mutations in the Drosophila homologues of the human Neurofibromatosis 2 and yeast CDC42 genes using a simple and efficient reverse-genetic method. Genetics 146: 245–252. 9136014 10.1093/genetics/146.1.245 PMC1207939

[25] Lee T, Luo L (1999) Mosaic analysis with a repressible cell marker for studies of gene function in neuronal morphogenesis. Neuron 22: 451–461. 10197526 10.1016/s0896-6273(00)80701-1

[26] Serpe M, Umulis D, Ralston A, Chen J, Olson DJ, et al (2008) The BMP-binding protein Crossveinless 2 is a short-range, concentration-dependent, biphasic modulator of BMP signaling in Drosophila. Dev Cell 14: 940–953. 18539121 10.1016/j.devcel.2008.03.023 PMC2488203

[27] Teleman AA, Cohen SM (2000) Dpp gradient formation in the Drosophila wing imaginal disc. Cell 103: 971–980. 11136981 10.1016/s0092-8674(00)00199-9

[28] Baron M, O'Leary V, Evans DA, Hicks M, Hudson K (2000) Multiple roles of the Dcdc42 GTPase during wing development in Drosophila melanogaster. Mol Gen Genet 264: 98–104. 11016838 10.1007/s004380000287

[29] Genova JL, Jong S, Camp JT, Fehon RG (2000) Functional analysis of Cdc42 in actin filament assembly, epithelial morphogenesis, and cell signaling during Drosophila development. Dev Biol 221: 181–194. 10772800 10.1006/dbio.2000.9671

[30] Araujo H, Negreiros E, Bier E (2003) Integrins modulate Sog activity in the Drosophila wing. Development 130: 3851–3864. 12835400 10.1242/dev.00613

[31] Wilcox M, DiAntonio A, Leptin M (1989) The function of PS integrins in Drosophila wing morphogenesis. Development 107: 891–897. 10532829 10.1242/dev.107.4.891

[32] Bement WM, Miller AL, von Dassow G (2006) Rho GTPase activity zones and transient contractile arrays. Bioessays 28: 983–993. 16998826 10.1002/bies.20477 PMC4364130

[33] Yan J, Lu Q, Fang X, Adler PN (2009) Rho1 has multiple functions in Drosophila wing planar polarity. Dev Biol 333: 186–199. 19576201 10.1016/j.ydbio.2009.06.027 PMC2728161

[34] Dominguez-Gimenez P, Brown NH, Martin-Bermudo MD (2007) Integrin-ECM interactions regulate the changes in cell shape driving the morphogenesis of the Drosophila wing epithelium. J Cell Sci 120: 1061–1071. 17327274 10.1242/jcs.03404

[35] Chen J, Honeyager SM, Schleede J, Avanesov A, Laughon A, et al (2012) Crossveinless d is a vitellogenin-like lipoprotein that binds BMPs and HSPGs, and is required for normal BMP signaling in the Drosophila wing. Development 139: 2170–2176. 22573617 10.1242/dev.073817 PMC3357910

[36] Bunt S, Hooley C, Hu N, Scahill C, Weavers H, et al (2010) Hemocyte-secreted type IV collagen enhances BMP signaling to guide renal tubule morphogenesis in Drosophila. Dev Cell 19: 296–306. 20708591 10.1016/j.devcel.2010.07.019 PMC2941037

[37] Wang X, Harris RE, Bayston LJ, Ashe HL (2008) Type IV collagens regulate BMP signalling in Drosophila. Nature 455: 72–77. 18701888 10.1038/nature07214

[38] Pastor-Pareja JC, Xu T (2011) Shaping cells and organs in Drosophila by opposing roles of fat body-secreted Collagen IV and perlecan. Dev Cell 21: 245–256. 21839919 10.1016/j.devcel.2011.06.026 PMC4153364

[39] Murray MA, Fessler LI, Palka J (1995) Changing distributions of extracellular matrix components during early wing morphogenesis in Drosophila. Dev Biol 168: 150–165. 7883070 10.1006/dbio.1995.1068

[40] Vuilleumier R, Springhorn A, Patterson L, Koidl S, Hammerschmidt M, et al (2010) Control of Dpp morphogen signalling by a secreted feedback regulator. Nat Cell Biol 12: 611–617. 20453847 10.1038/ncb2064

[41] Szuperak M, Salah S, Meyer EJ, Nagarajan U, Ikmi A, et al (2011) Feedback regulation of Drosophila BMP signaling by the novel extracellular protein larval translucida. Development 138: 715–724. 21266407 10.1242/dev.059477

[42] Kesavan G, Sand FW, Greiner TU, Johansson JK, Kobberup S, et al (2009) Cdc42-mediated tubulogenesis controls cell specification. Cell 139: 791–801. 19914171 10.1016/j.cell.2009.08.049

[43] Lahoz A, Hall A (2008) DLC1: a significant GAP in the cancer genome. Genes Dev 22: 1724–1730. 18593873 10.1101/gad.1691408 PMC2732422

[44] Massague J (2008) TGFbeta in Cancer. Cell 134: 215–230. 18662538 10.1016/j.cell.2008.07.001 PMC3512574

