# Supplementary figures and images for: A Feed-Forward Loop Coupling Extracellular BMP Transport and Morphogenesis in Drosophila Wing
Source: PLoS Genet. 2013 Mar 21;9(3):e1003403. doi: 10.1371/journal.pgen.1003403 (PMC3605110; doi:10.1371/journal.pgen.1003403)

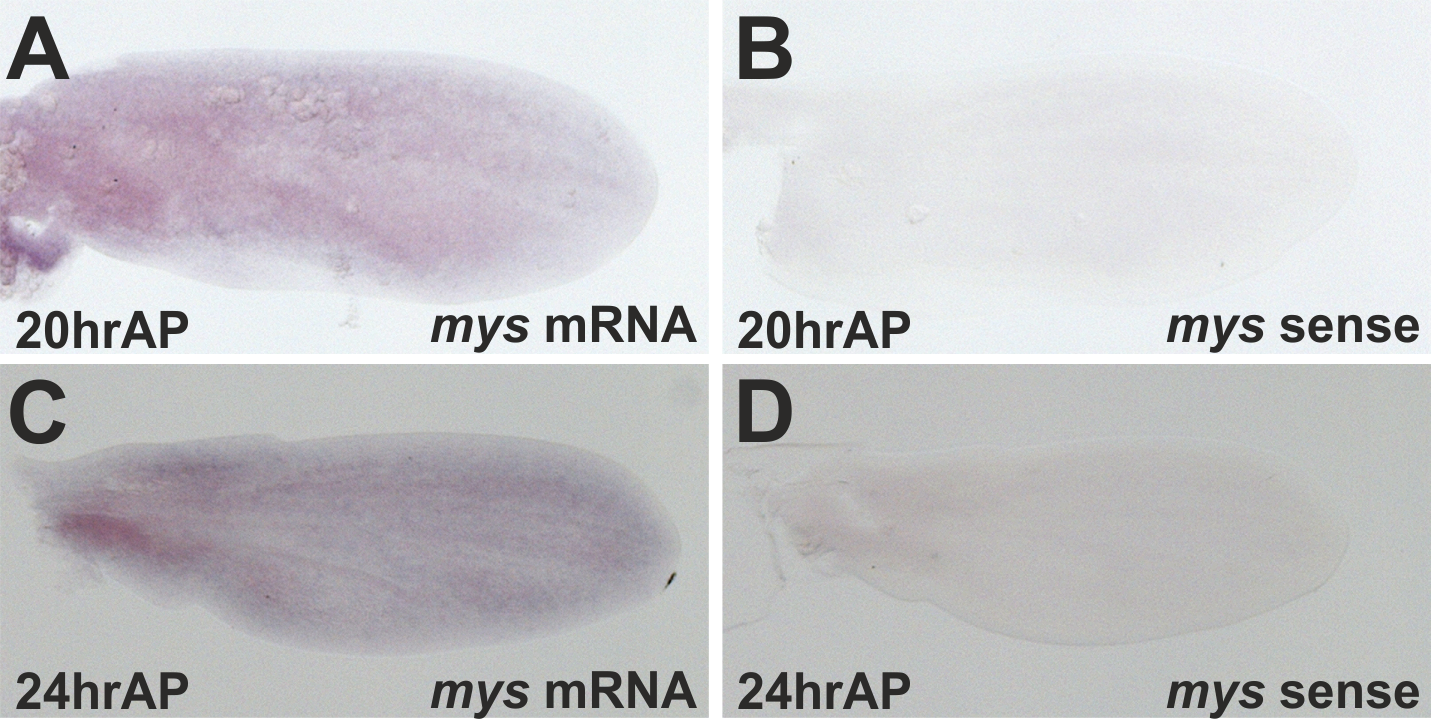

Supplement: Figure S1 — ß-integrin expression in the pupal wing. (A—D) in situ hybridization of ß-integrin at 20 hr AP (A, B) and at 24 hr AP (C, D). Antisense (A, C) and sense (B, D) probes of myospheroid (mys) encoding ß-integrin. (TIF) [file pgen.1003403.s001.tif]

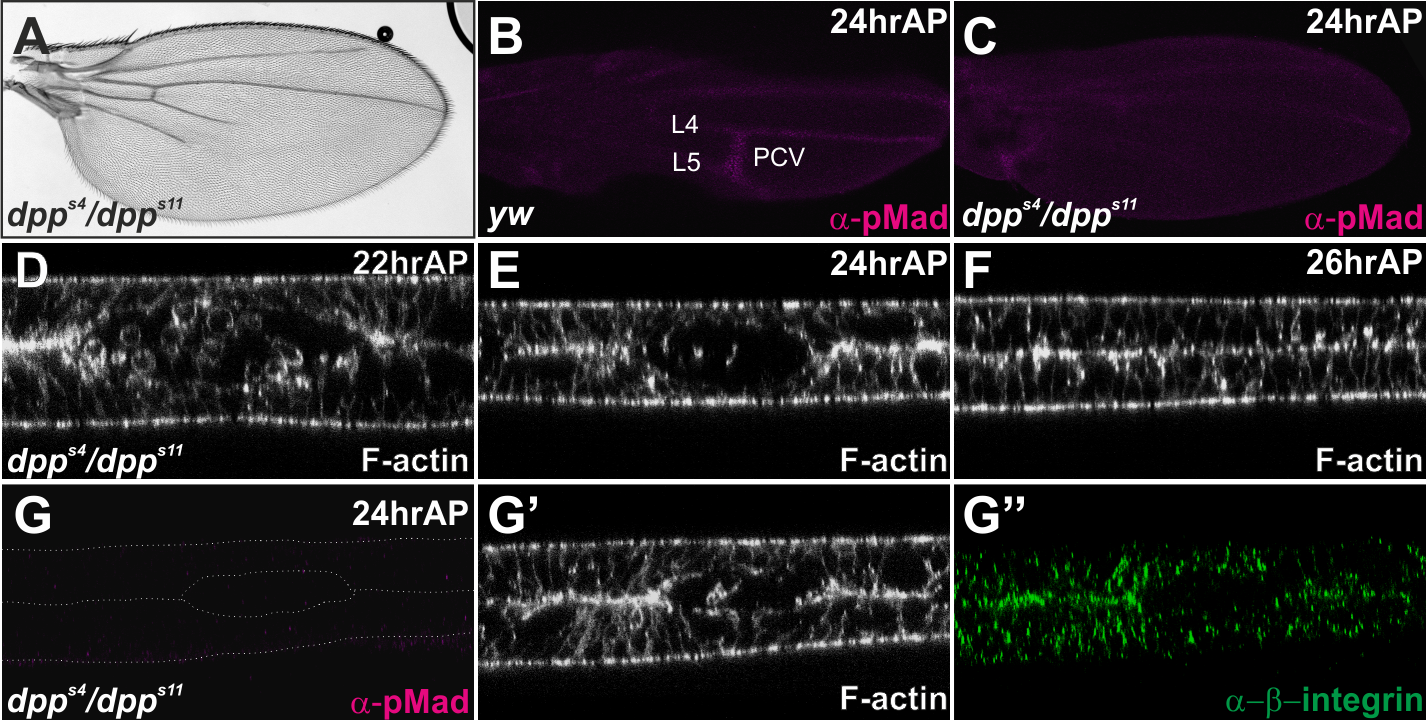

Supplement: Figure S2 — The initial PCV morphogenesis in dpps4/dpps11. (A) Adult wing of dpps4/dpps11. (B, C) pMad staining at 24 hr AP in control yw (B) and in dpps4/dpps11 (C). (D–F) Optical cross-sections of the PCV region. F-actin staining at 22 hr AP (D), 24 hr AP (E), 26 hr AP (F) in dpps4/dpps11. A number of hemocytes were observed in the lumen at 22 hr AP. Slight delay of apposition of the two wing layers may reflect loss of the LVs fates in dpps4/dpps11. (G–G″) Optical cross-sections of the PCV region. pMad (G), F-actin (G′), and ß-integrin (G″) staining at 24 hr AP in dpps4/dpps11. (TIF) [file pgen.1003403.s002.tif]

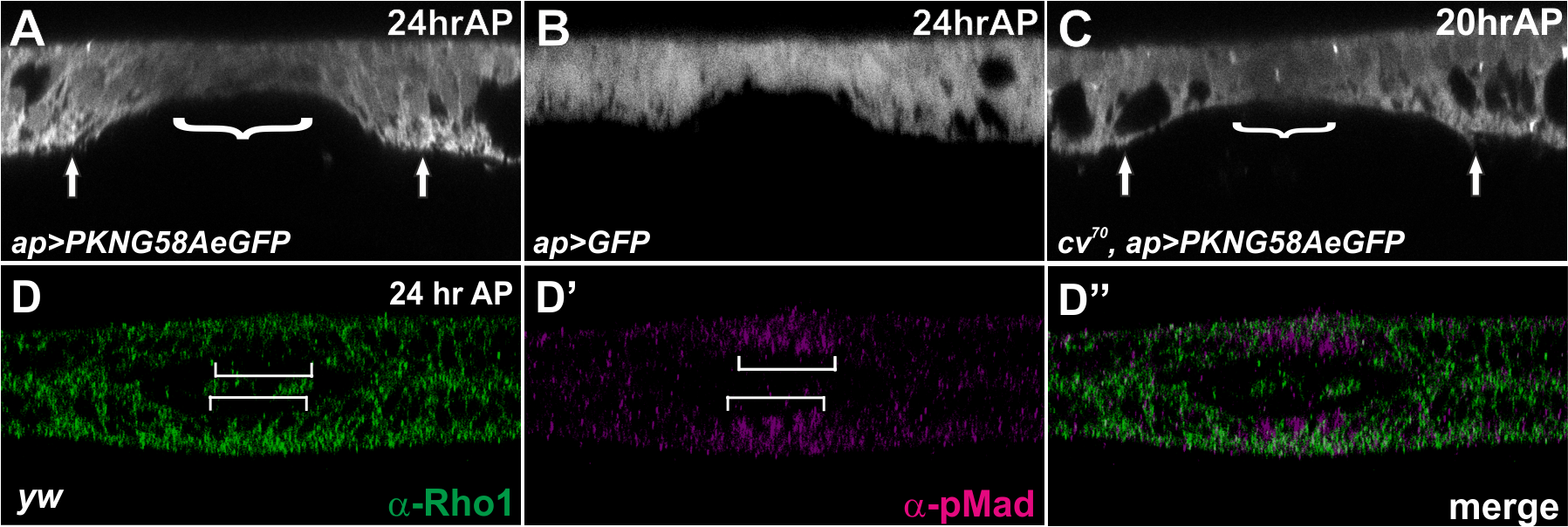

Supplement: Figure S3 — Rho1 activity and protein localization around the PCV region. (A) Rho1 activity in the dorsal layer at 24 hr AP in ap>PKNG58AeGFP. (B) GFP signal in the dorsal layer of shv>GFP. (C) Rho1 activity in the dorsal wing layer at 20 hr AP in cv70, ap>PKNG58AeGFP. (A, C) The basal side of the invervein regions is indicated by arrows. The PCV region is marked by parentheses. ap-Gal4 is induced in the dorsal layer of the wing epithelial cells. (D) Rho1 (D), pMad (D′) staining, and merge (D″) in wild-type yw. All the images are optical cross-sections of the PCV region. (TIF) [file pgen.1003403.s003.tif]

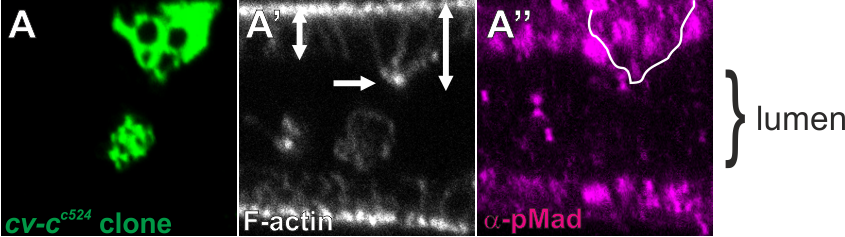

Supplement: Figure S4 — Cell-autonomous F-actin accumulation at the basal side of the PCV region in cv-cc524 clones. A sagittal section of the PCV region, containing cv-c null clones. (A) cv-cc524 clones (green), (A′) F-actin (white), and (A″) pMad (purple). F-actin accumulation at the basal side of the PCV is indicated by arrow. Apical-basal cell lengths of wild-type and cv-c mutant are indicated by double-headed arrows. (TIF) [file pgen.1003403.s004.tif]

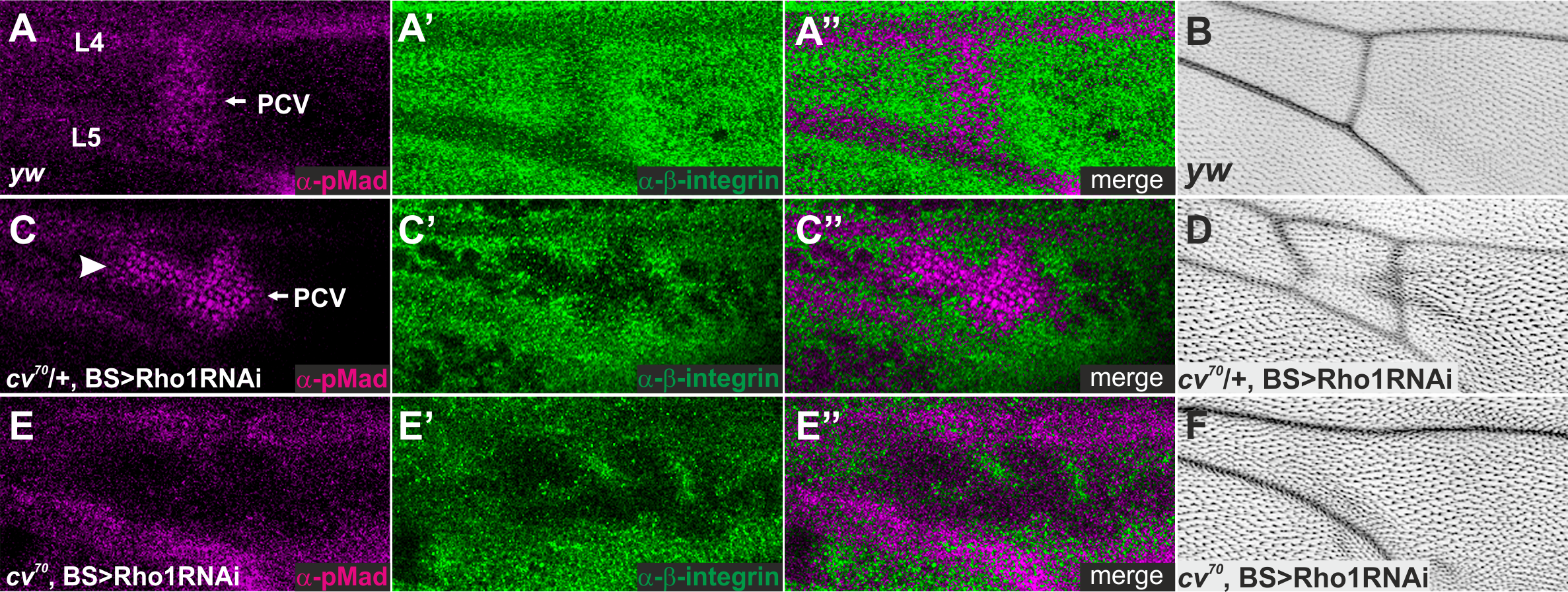

Supplement: Figure S5 — Loss of Rho1 by RNAi induces Sog-Cv-dependent BMP signaling. pMad (A, C, E), ß-integrin (A′, C′, E′) staining, merged images (A″, C″, E″), and adult wings (B, D, F) in wild-type yw (A—A″, B), cv70/+, BS1348>Rho1 RNAi (C—C″, D), and cv70, BS1348>Rho1 RNAi. (E—E″, F). The PCV position is indicated by arrows (A, C). Ectopic pMad accumulation is marked by arrowhead (C). All the pupal wings were fixed at 24 hr AP. Images were processed by maximum intensity profile from a single wing layer. (TIF) [file pgen.1003403.s005.tif]

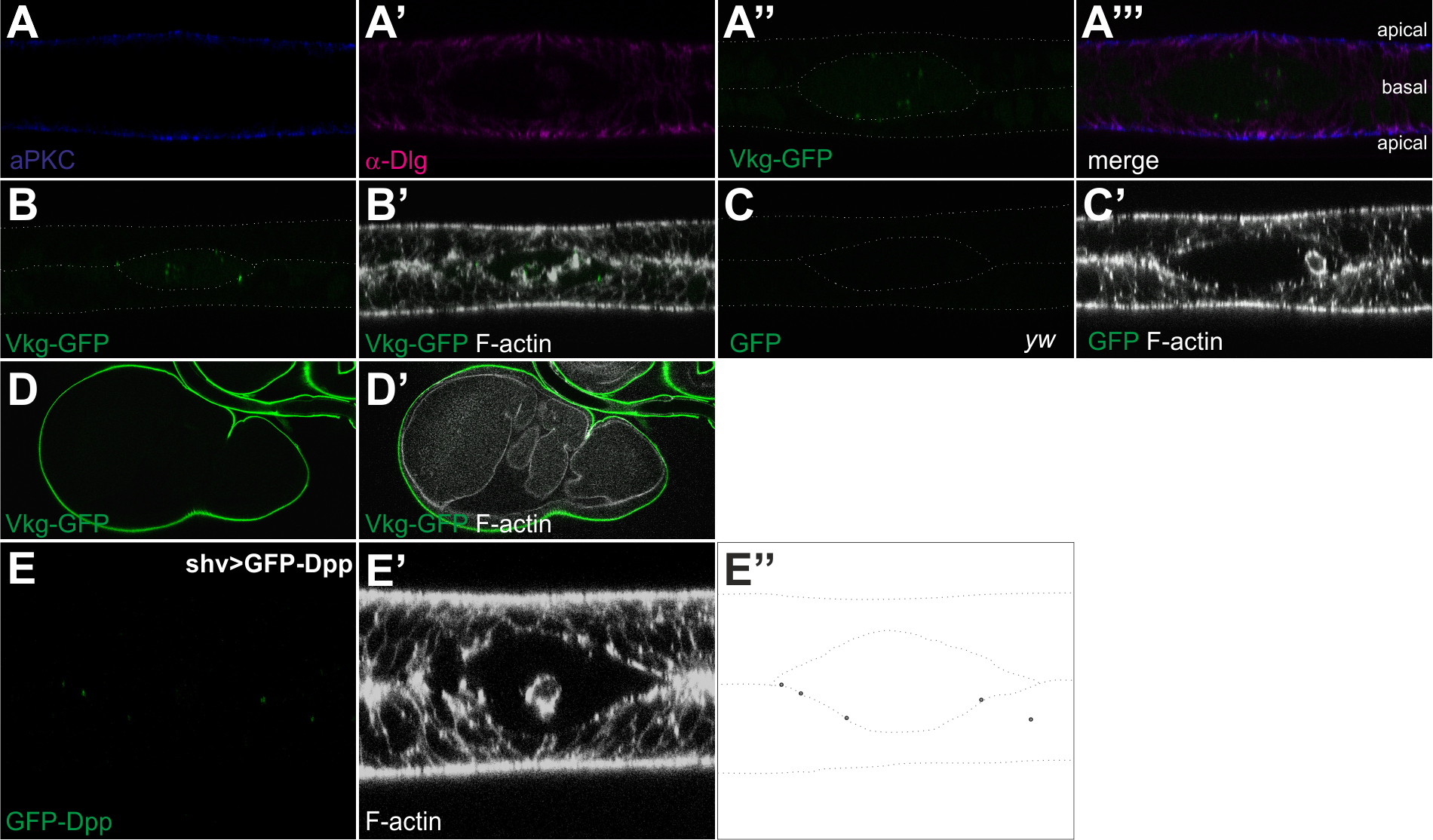

Supplement: Figure S6 — Apical-basal polarity in the PCV region and intervein region. (A–C) Optical cross-sections of the PCV region at 24 hr AP. (A) aPKC staining (A), Dlg staining (A′), Vkg-GFP signal (A″), and merged image (A‴) in Vkg-GFP wing. (B) Vkg-GFP signal (B) and F-actin staining (B′) in Vkg-GFP pupal wing. (C) GFP signal (C) and F-actin staining (C′) in control yw pupal wing. In addition to few punctuate signal, Collagen IV was also weakly and uniformly distributed in the lumen. (D) Vkg-GFP signal (D) and F-actin staining (D′) in Vkg-GFP larval wing imaginal disc. (E) Optical cross-sections of the PCV region at 24 hr AP. Representative images of GFP-Dpp distribution along apical-basal axis. GFP-Dpp dots (E), F-actin staining (E′), and schematic position of GFP-Dpp dots (E″) in shv>GFP-Dpp. (TIF) [file pgen.1003403.s006.tif]
